# TUBB4B Downregulation Is Critical for Increasing Migration of Metastatic Colon Cancer Cells

**DOI:** 10.3390/cells8080810

**Published:** 2019-08-01

**Authors:** Katarzyna Sobierajska, Wojciech M. Ciszewski, Marta E. Wawro, Katarzyna Wieczorek-Szukała, Joanna Boncela, Izabela Papiewska-Pajak, Jolanta Niewiarowska, M. Anna Kowalska

**Affiliations:** 1 Department of Molecular Cell Mechanisms, Medical University of Lodz, 92215 Lodz, Poland; 2 Department of Endocrinology and Metabolic Diseases, Medical University of Lodz, 93338 Lodz, Poland; 3 Institute of Medical Biology, PAS, 93232 Lodz, Poland

**Keywords:** microtubules, vimentin, colon cancer, EMT, migration

## Abstract

Tumor metastasis, the major problem for clinical oncology in colon cancer treatment, is linked with an epithelial-mesenchymal transition (EMT). The observed cellular transformation in this process is manifested by cell elongation, enhanced cell migration and invasion ability, coordinated by cytoskeleton reorganization. In the present study, we examined the role of tubulin-β4 (TUBB4B) downregulation that occurs during EMT in colon cancer cells, in the modulation of the function of microtubules. Based on biochemical and behavioral analysis (transmigration) we posit that the decrease of the TUBB4B level is critical for microtubule-vimentin interaction and contributes to the maintenance of polarity in migrating cells. The microscopic studies revealed that TUBB4B decrease is accompanied by cell elongation and increased number of matured focal adhesion sites, which is a characteristic of the cell metastatic stage. We also demonstrated faster polymerization of microtubules in cells with a lower level of TUBB4B. Simultaneous TUBB3 upregulation, reported during EMT, acts additively in this process. Our studies suggest that the protein level of TUBB4B could be used as a marker for detection of the preinvasive stages of the colon cancer cells. We also concluded that chemotherapy enriched to increase TUBB4B level and/or to stabilize microtubule polymerization might more effectively prevent metastasis in colon cancer development.

## 1. Introduction

Colon cancer is the third most cancer commonly diagnosed cancer worldwide. Despite widespread screening, it is frequently diagnosed at metastatic stages and remains the second leading cause of cancer-related death [1]. A critical process associated with the tumor metastasis is the epithelial-mesenchymal transition (EMT) of the cancer cells [2]. During EMT, cells are transformed from cobblestone apical-basal polarized epithelial cells to spindle-like mesenchymal cells. Modulations observed in cancer cell morphology are the effect of cytoskeleton remodeling. The complex of filaments composed of microfilaments, microtubules, and intermediated filaments are connected to each other via the proteins interacting with them, causing the cytoskeleton to be seen as a single functional unit. In contrast to the well-described role of actin microfilaments in mesenchymal transdifferentiation [3], the relevance of microtubules and intermediate filaments during EMT is not fully understood. 

Microtubules are composed of the dimers of α- and β-tubulins (TUBAs and TUBBs). In mammalian cells, there are eight TUBA and seven TUBB, and their particular expression depends on cell type [4,5]. Alterations of various tubulin levels are observed in pathological conditions [6,7]. Microtubules constantly undergo polymerization and depolymerization in the phenomenon called “dynamic instability” and in vitro experiments have demonstrated that the composition of tubulin subunits in microtubules regulates the speed of this process [4]. Additionally, alterations of posttranslational modifications within tubulin subunits, such as phosphorylation, might regulate the interaction between microtubules and microtubule-associated proteins (MAPs), therefore affecting the dynamic instability of microtubules [8]. In the interphase cells, microtubules are located near nucleus, in the centrosome (minus end) and radiate toward the cell periphery (plus end) thus maintaining the cell shape and regulating cell-cell interaction, cell-matrix adhesion, protein transport, and cell movement [9,10]. Further, microtubules form the mitotic spindle that enables correct chromosomal segregation during cell division. Hence, they are considered one of the main targets for cancer therapy intended to block cell proliferation [11]. Changes of TUBB3 and TUBB4B levels have been studied with respect to the regulation of cancer progression [6]. Understanding of the well-characterized mechanism of decreased sensitivity of cancer cells to tubulin-binding agents (TBAs) like vinca alkaloids and taxanes, comes from the analysis of TUBB3 and TUBB4B level regulation in non-small cell lung cancer and prostate cancer development [12,13,14,15,16]. Additionally, TUBB3, TUBB4B, and TUBB6 downregulation were observed in taxane-resistant breast cancer cells [17].

Microfilaments and microtubules are polymers of single types of proteins. In contrast, intermediate filaments are composed of several proteins whose expression is dependent on types of cells. Among 67 proteins that belong to the intermediate filament in humans, only vimentin, the metastasis marker, is associated with enhanced cell migratory properties [18]. However, vimentin-dependent molecular mechanisms involved in enhanced cell migration during EMT remain largely elusive. In contrast to other intermediate filament proteins (keratin, desmin) which are observed primarily in the rear and perinuclear region of the cells, vimentin is mainly located in lamellipodia and is critical for their formation [19]. Filamentous vimentin is detected near large focal adhesion, whereas non-filamentous vimentin or other intermediate filaments proteins are located in the vicinity of the small focal adhesions [20]. Hence, assembly states of vimentin seem not only to affect lamellipodia formation but may also be involved in establishing the anisotropy of focal contacts and focal adhesions and in consequence, seem to be required for increased migration of tumor metastatic cells. Further, the interaction of vimentin with microtubules regulates the polarity of the cytoskeleton of the cells [21].

The molecular mechanism and the role of particular tubulins in the EMT process are still unknown. Therefore, we focused on the role of TUBB4B downregulation and TUBB3 modulation in microtubule polymerization during EMT in colon cancer progression. It has been known that tumor growth factor-beta 1 (TGF-β1) and Snail are the main stimulators of EMT. We used a model of pre-invasive colon cancer cell (CRC) lines (HT-29 and LS-180) with EMT induced by either TGF-β1 stimulation or by stable expression of the Snail transcriptional factor. Based on our results, we conclude that microtubule-intermediate filament interactions are critical for the EMT process in colon cancer and that therapies based on microtubule reorganization supplemented with chemotherapeutics directed against vimentin might be more effective in cancer treatment in the invasive stages.

## 2. Materials and Methods

### 2.1. Reagents

All reagents were obtained from Sigma-Aldrich unless stated otherwise. The tissue culture reagents, including McCoy’s 5A and DMEM medium, fetal bovine serum (FBS), were from Invitrogen (Eggenstein, Germany). Cell Line Nucleofector^®^Kit R was from Lonza (Allendale, NJ, USA). TriPure Reagent, LightCycler 480 SYBR Green I Master PCR reaction mix, PhosStop phosphatase inhibitor, and cOmplete Protease Inhibitor Cocktail were from the Roche Diagnostics Corporation (Indianapolis, ID, USA). All primers were made by Genomed (Warsaw, Poland). M-MLV Reverse Transcriptase was purchased from Promega Corp. (Madison, WI, USA). Enhanced Chemiluminescence (ECL) Western blotting substrate, M-PER Extraction Reagents, NE-PER Nuclear and the BCA Protein Assay Kit, Moloney Murine Leukemia Virus Reverse Transcriptase were from Thermo Scientific Pierce (Minneapolis, MN, USA). Rabbit anti-vinculin antibodies were from Cell Signaling (Danvers, MA, USA), goat anti-mouse antibodies, anti-rabbit antibodies, and mouse anti-GAPDH conjugated with horseradish peroxidase were purchased from Santa Cruz Biotech (Santa Cruz, CA, USA). 

### 2.2. Cell Culture and Preparation

Cancer cell HT-29 and LS180 lines, isolated from pre-invasive stage of primary colon adenocarcinoma, and LoVo cells isolated from patient with colon cancer in invasive stages, were purchased from the American Type Culture Collection (Rockville, MD, USA) and were cultured in McCoy’s (HT-29) or MEM-α (LS180) medium supplemented with l-glutamine (2 mM), 10% (*v*/*v*) fetal bovine serum (FBS), streptomycin (100 μg/mL), and penicillin (100 units/mL). The cells were maintained as a monolayer (37 °C; humidified atmosphere with 5% CO_2_). Previously characterized culture of stable Snail-transfected HT-29 cell (clone 3 and clone 8) and LS180 (clone 2 and clone 5) [22,23] were also supplemented with 100 μg/mL (HT-29) or 75 μg/mL (LS180) geneticin.

### 2.3. siRNA Assay

HT-29 cells were transfected by electroporation as described elsewhere [22] with the mix of four siRNAs sequence targeting human TUBB4B (concentration of 100 nM according to previous studies) or scramble sequences of siRNA (negative control) from Dharmacon (Lafayette, CO, USA). Then, cells were silenced for 48 h prior to further analysis as described previously [22]. Additionally, the specificity of TUBB3 silencing was analyzed by the evaluation of the expression level of other tubulin subunits (TUBB1, TUBB3, TUBB4B, TUBB6).

### 2.4. Confocal Microscopy

First, 5 × 10^4^ cells/mL were seeded and cultured on slides (37 °C in a humidified atmosphere of 5% CO_2_; 24 h). Next, cells were washed with PBS, fixed with 4% formaldehyde in PHEM buffer (25 mM Hepes, 60 mM Pipes, 10 mM EGTA, 4 mM MgCl_2_, 0.1 mM EDTA pH 6.9, and with 1 mg/mL aprotinin, 1 mM leupeptin, and 1 mM PMSF; 20 min; RT) and prepared as described previously [24]. Then, after PHEM buffer washing cells were permeabilized with 0.1% Triton X-100 (*v*/*v*) and non-specific sites were blocked with 2% (*v*/*v*) BSA in PHEM buffer (60 min; RT). The cellular proteins were labeled by antibodies at (60 min; 37 °C) and the nucleus with DAPI (30 min; RT). Finally, the images were scanned and captured with a Leica TCS SP8 laser scanning confocal microscope (63×; 1.4 NA objective) (Leica Microsystems GmbH, Mannheim, Germany). For the analysis of fluorescence intensity, at least 50 cells from each experiment were analyzed using the LAS AF software v. 3.3.0.10134 (Leica Microsystems) or ImageJ software (mean intensity from at least 50 chosen cells) [25]. Additionally, focal adhesion area and numbers of that structures (labeled by anti-vimentin and additionally with anti-filamin and anti-talin antibodies) were analyzed in 50 randomized chosen cells in ImageJ software as the mean intensity of the region of interest.

### 2.5. Cytoskeleton Isolation

Microtubules fraction was isolated from the cells as described previously [26]. Briefly, cell pellets were homogenized in PB buffer (0.1 M K-PIPES (pH 6.8), 0.5 mM MgCl_2_, 2 mM EGTA, 0.1 mM EDTA, 0.1% (*v*/*v*) β-mercaptoethanol, 1 mM ATP with PhosStop phosphatase inhibitor and cOmplete Protease Inhibitor Cocktail) and centrifuged (100,000× *g*; 60 min; 4 °C). The supernatants containing cytosolic tubulins were collected, mixed with a half volume of 100% glycerol (preheated at 37 °C and supplemented with ATP and 3.5 mM MgCl_2_), and incubated to polymerize (60 min; 37 °C). After centrifugation (100,000× *g*; 45 min; 37 °C) the pellet containing microtubules was collected, resuspended in PBS (ice-cold) and incubated to depolymerize (30 min; on ice). Tubulin cleaning by alternating polymerization and depolymerization was repeated twice. 

### 2.6. Polymerization Assay

In brief, tubulin proteins isolated as described in Section 2.5 above and suspended in a 96-well plate in G-PEM buffer (80 mM PIPES, 2 mM MgCl_2_, 0.5 mM ethylenediaminetetraacetic acid, 1.0 mM GTP (pH 6.9), and 5% glycerol). The absorbance was measured at 340 nm from 0 to 80 min (Synergy H4 multimode microplate reader BioTek, Winooski, VT, USA). The assay was performed in three independent experiments [27].

### 2.7. Immunoprecipitation of Microtubules

Immunoprecipitation of microtubules was performed as described previously [22]. Briefly, the detergent-insoluble fraction was centrifuged (15,000× *g*; 15 min; RT) and soluble extracts were collected for immunoprecipitation. After pre-cleaning, 500 μg of protein from each extract was incubated with 2 μg of rabbit anti-vimentin antibodies with a rotator (overnight; 4 °C). Next, 100 μL of protein A/G agarose beads were added to each extract, incubated (3 h; 4 °C), and washed three times with PBS. Finally, beads were suspended in 2× concentrated SDS-PAGE loading buffer and boiled (5 min). Proteins released from the resin were immediately separated by SDS-PAGE as described below.

### 2.8. Lysate Preparation and Western Blot

Cell extracts were prepared as described previously [28]. Briefly, exponentially growing cells were lysed with M-PER Mammalian Protein Extraction Reagent supplemented with Halt Protease Inhibitor Cocktail according to the manufacturer’s instructions. The concentration of proteins in each sample was quantified with the BCA Protein Assay Kit according to the manufacturer’s protocol. Protein lysates (30 µg), immunoprecipitate, or tubulin fraction extracts were separated on 10% SDS-PAGE gels (Bio-Rad, CA, USA) and electroblotted (120 min; 200 mA; 4 °C) onto nitrocellulose membranes (Bio-Rad). The membranes were blocked with 5% BSA in PBS (2 h; RT), incubated with primary antibodies (overnight; 4 °C), and after washing incubated with horseradish peroxidase-conjugated secondary antibodies (1 h; RT). Chemiluminescence detection proceeded with Pierce ECL Western Blotting Substrate and Kodak BioMax Light Film (Eastman Kodak, Rochester, NY, USA). Protein bands were scanned (HP Scanjet G4050) and quantified using Gel Doc 2000 gel documentation system (Bio-Rad).

### 2.9. RNA Isolation and Real-Time PCR

Total RNA was isolated from the cells using the TriPure reagent according to the manufacturer’s instructions. RNA transcription (1 μg) into cDNA was made with Moloney Murine Leukemia Virus Reverse Transcriptase according to the manufacturer’s instruction. Next, the expression levels of *TUBB4B* were evaluated by real-time quantitative PCR using the forward 5′-ATGAGGGAGATCGTGCAC-3′, and reverse 5′-TCCAGGACCGAATCCACCA -3′ primers. The analysis was made using a LightCycler (Roche Diagnostic) and normalized to the housekeeping glyceraldehyde 3-phosphate dehydrogenase (*GAPDH*) gene, expression of which was determined using following primers: forward 5′-GAGAGATGATGACCCTTTTGGC-3′ and reverse 5′-CCATCACCATCTTCCCAGGAGCG-3′. The controls of each experiment constituted samples without reverse transcription enzyme and with no template cDNA. The relative mRNA expression levels were estimated using 2^-ΔΔ^Ct quantification. Amplification of specific transcripts was further confirmed by obtaining melting curve profiles [29].

### 2.10. Cell Morphology Analysis

First, 5000/cm^2^ cells were seeded on the 6-well plate, and 24 h later, cells were treated according to procedure. After 2 days, cell morphology of semi-confluent culture was observed under the microscope and captured by an Olympus camera (Olympus, Olympus Optical Co., Ltd., Tokyo, Japan). The elongation ratio was calculated as described elsewhere [30].

### 2.11. Cell Migration Assay

Assays were performed as described previously [31] in modified Boyden chambers containing polycarbonate membranes (Transwell^®^; Costar, Cambridge, MA, USA). Briefly, the lower chambers were filled with 0.4 mL of medium containing 20 μg/mL collagen while 1 × 10^5^ cells were placed into the upper chamber. After 6 h (37 °C; 5% CO_2_) the migrated cells were fixed in 4% paraformaldehyde in PBS (20 min; RT) and non-migrated cells were wiped with a cotton-tip applicator from the upper surface. The cells were stained with crystal violet, photographed, and quantified with the ImageJ program.

### 2.12. Statistical Analyses

The results are presented as the mean of at least three independent experiments ± SD. Statistical significance of the differences between experimental conditions was determined by Student’s *t*-test for unpaired groups or ANOVA followed by Tukey’s test (GraphPad Prism, CA, USA). *p* < 0.05 (*), *p* < 0.01 (**), or *p* < 0.005 (***) was considered statistically significant.

## 3. Results

### 3.1. TUBB4B Is Downregulated during EMT 

**Figure 1:**
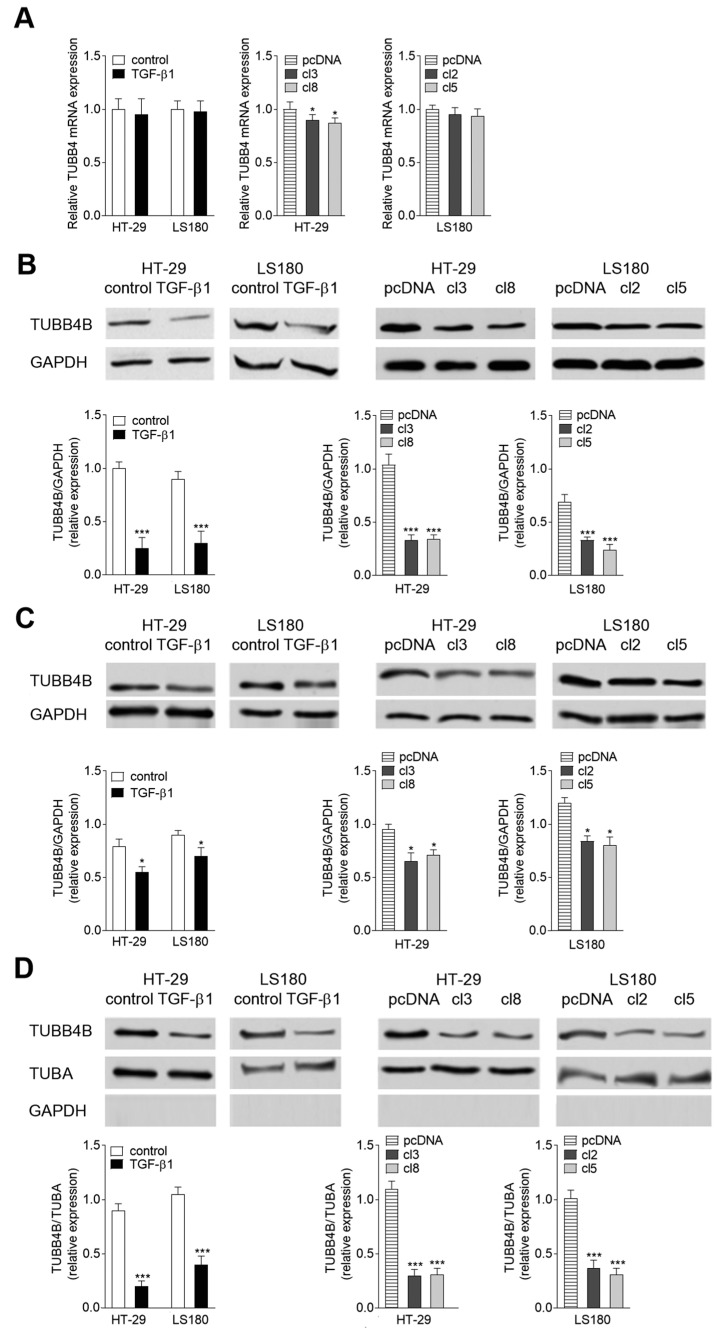
Induction of epithelial-mesenchymal transition (EMT) in colon cancer cell lines results in TUBB4B downregulation. (**A**) The expression of tubulin-β4 (TUBB4B) was determined in controls, TGF-β1-treated HT-29 and LS180 colon cancer cell (left panel), empty vector- and stable Snail-transfected HT-29 (cl3, cl8) (middle panel) and LS180 (cl2, cl5) clones (right panel). The relative mRNA level of TUBB4B was normalized to glyceraldehyde 3-phosphate dehydrogenase (GAPDH). (**B**) The protein levels in the whole cell lysates were evaluated by Western blot assay using a mouse monoclonal anti-TUBB4B antibody. The quantity of the TUBB4B protein was normalized to GAPDH. (**C**) Equal amounts of cytosol and (**D**) cytoskeleton cell fractions were analyzed by Western blot using monoclonal mouse anti-TUBB4B antibodies. Protein level was normalized to GADPH for the cytosol (**C**) and to alpha-tubulin (TUBA) for the cytoskeleton (**D**). Additionally, to confirm the purity of cytoskeleton fraction isolation, the presence of GAPDH was analyzed (**D**). Statistical variability was measured relative to control (left panel **B**,**C**,**D**) or stable pcDNA3.1 plasmid-transfected cells (right panel **B**,**C**,**D**); *n* = 3; * *p* < 0.05, *** *p* < 0.005.

Our previous studies revealed that TUBB4B protein level was reduced in a HT-29 CRC line overexpressing Snail [22]. To follow the hypothesis that TUBB4B is downregulated during mesenchymal transdifferentiation, we analyzed the expression of beta-tubulins in two EMT cellular models (Figure 1): HT-29 and LS180 cell lines stimulated by TGF-β1 (5 ng/mL for 48 h) [22] or overexpressed transcriptional factor Snail (HT-29/Snail and LS180/Snail). In agreement with our early observations, statistically significant mRNA downregulation (0.88 of control) of TUBB4B was detected by real-time PCR assay of HT-29/Snail clones (Figure 1A). However, the downregulation of mRNA was neither observed during TGF-β1 stimulation nor in LS180/Snail clones.

The tubulin level modulations were also measured by Western blots. We observed a strong decrease of TUBB4B protein level in both HT-29 and LS180 cell lines undergoing EMT. Approximately 0.75 downregulation of TUBB4B was found in the HT-29 (TGF-β1-treated and Snail clones) (Figure 1B) and slightly less in LS180 cells (0.65 decrease in comparison to control, unstimulated cells). We also analyzed the changes of the TUBB4B levels in cytosol (Figure 1C) and cytoskeleton (Figure 1D) fractions of non-stimulated, pre-invasive HT-29 and LS180 cells. GAPDH protein level was evaluated and served as the loading control (cytosol) or a purity control (cytoskeleton). Total intensity of alpha-tubulin (TUBA) was used for normalization in cytoskeleton fraction. We found a higher level of TUBB4B in cytosol components isolated from non-stimulated, pre-invasive HT-29 and LS180 cell lines and from both cell lines transfected with empty vector than in cells after EMT induction both for TGF-β1-treated and Snail-expressing clones (Figure 1C). Downregulation of TUBB4B after TGF-β1 stimulation was even more markedly observed in cytoskeleton (Figure 1D) of HT-29 cells and LS180 cells (0.8 and 0.6 down as compared to control, non-stimulated cells, respectively). The same phenomenon was observed in both CRC lines transfected with Snail. Similarly, to the case of TGF-β1 stimulation, the levels of TUBB4B were dropping down more in HT-29 than in LS180 (0.75 and 0.65 decrease, respectively as compared to cells transfected with pcDNA). Further, we examined TUBB4B expression in LoVo cells isolated from invasive stages of colon cancer [22]. This line characterizes with a higher basal expression of Snail as compared with preinvasive cell lines HT-29 and LS180 [22]. Immunochemical analysis of the lysates from HT-29, LS180 and LoVo cell lines demonstrated a negative correlation between the expression of Snail and TUBB4B protein levels (Figure S1).

### 3.2. Phosphorylation and Glycosylation of TUBB4B Are Not Observed in EMT

**Figure 2:**
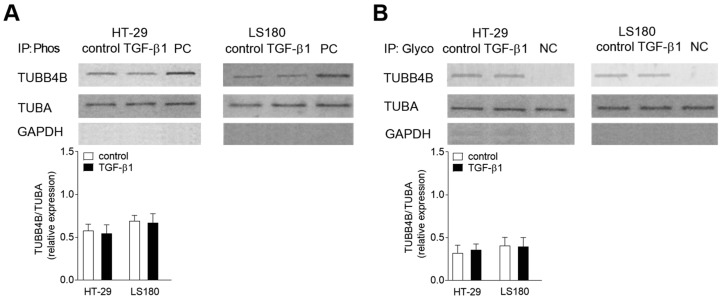
Posttranslational modifications of TUBB4B are not observed in EMT in colon cancer cells. Phosphorylation (Phos) and glycosylation (Glyco) levels in the cytoskeleton. Lysates of control and TGF-β1-treated HT-29 and LS180 cells were immunoprecipitated with antibodies recognizing phosphorylated (**A**) or glycosylated (**B**) residues, followed by Western blot assay with antibody binding to TUBB4B. GAPDH was used as the purity control, whereas alpha-tubulin (TUBA) level as the cytoskeleton loading control. Error bars represent the SD from three independent experiments (*n* = 3). Statistical variability was measured relative to control samples. The positive control (PC) of TUBB4B phosphorylation was a control cell treated with Phosphatase Inhibitor Cocktail1-4 (Sigma-Aldrich). The negative control (NC) of TUBB4B glycosylation was a control cells treated with tunicamycin. *n* = 3.

Further, we assessed the posttranslational modifications of TUBB4B in colon cancer cells undergoing EMT. We evaluated two modifications within this tubulin subunit located in the microtubules—phosphorylation (Figure 2A) and glycosylation (Figure 2B). The alterations within TUBB4B were examined in the microtubular fraction by immunoprecipitation assay of serine residues with P-Ser antibody recognizing phosphorylated proteins, or an antibody that binds to glycolyzed fragment of each protein. This was followed by Western blot with an antibody specifically recognizing TUBB4B. We also analyzed the level of TUBA in the microtubular fraction before immunoprecipitation as a control for the total protein content of the experiment. Additionally, GAPDH levels were evaluated to check the purity of isolated microtubular fraction. Surprisingly, our studies revealed that stimulation by TGF-β1 did not induce any changes in the level of TUBB4B phosphorylation (Figure 2A) or glycosylation (Figure 2B) in both colon cancer cell lines HT-29 and LS180 studied. As a positive control (PC) of phosphorylation modulation, we used lysate from the cells treated with a mix of Phosphatase Inhibitor Cocktail 1–4 (according to manufacturer’s instruction) which block protein dephosphorylations. Negative control (NC) of glycosylation was the cell lysate treated with tunicamycin that blocked glycosylation of tubulin in microtubules. 

### 3.3. TUBB4B Level Affect Cell Morphology and Microtubule Location in TGF-β1 Stimulated HT-29 Cells 

**Figure 3:**
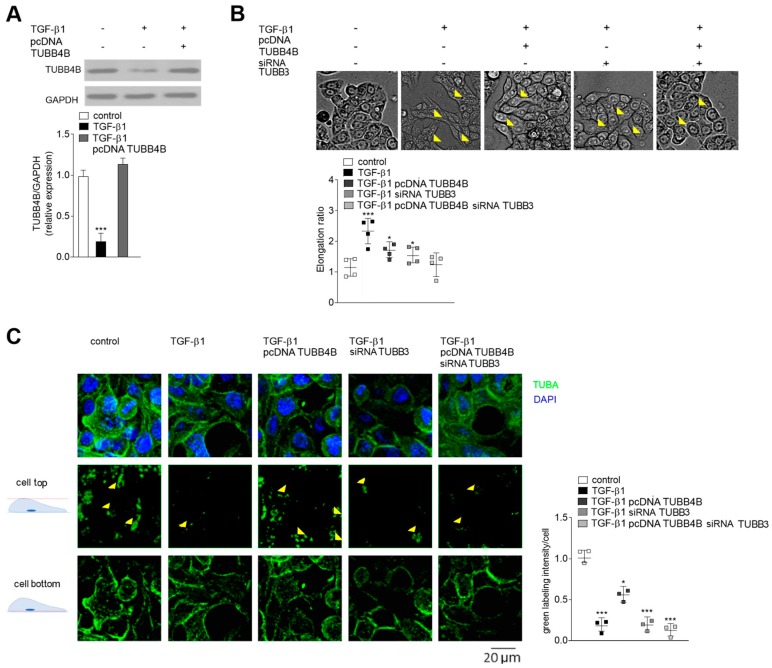
Downregulation of TUBB4B in TGF-β1 treated HT-29 cells affects cell morphology and microtubule polarization. (**A**) The extent of TUBB4B overexpression in control and TGF-β1-treated HT-29 cells was analyzed by Western blot assay using a monoclonal mouse anti-TUBB4B antibody. GAPDH was used as a control. (**B**) Morphology was determined after two days of HT-29 cell stimulation by TGF-β1 in the absence or presence of pcDNA-TUBB4B or siRNA-TUBB3. The yellow arrows indicate the places where the cells did not connect with each other suggesting disruption of the cell-cell contacts; *n* = 4; * *p* < 0.05, *** *p* < 0.005. (**C**) The effect of TUBB4B overexpression and/or TUBB3 silencing on microtubule localization in the HT-29 cells was analyzed by immunocytochemical studies. Anti-alpha-tubulin A (green channel), and DAPI (blue channel). The analysis of mean value of green labeling was quantified in ImageJ software and is presented as intensity per cell in 50 randomly chosen cells. Statistical significance is assessed relative to control cells. Error bars represent the SD from three independent experiments; *n* = 3; * *p* < 0.05, *** *p* < 0.005. + with and – without as indicated in the Figure.

In the next step of our studies, we focused on understanding the role of TUBB4B during EMT (Figure 3). Since stimulation by TGF-β1 reduced the level of TUBB4B, we restored it by transient transfection of the TGF-β1-stimulated HT-29 cells with a vector carrying TUBB4B (pcDNA-TUBB4B; Figure 3A). The microscopic studies revealed that HT-29 cells stimulated by TGF-β1 become elongated but after TUBB4B expression, this process was reversed, although not completely (Figure 3B). Calculated elongation ratio was 2.3 times higher in TGF-β1 stimulated cells and 1.7 times higher in TUBB4B expressing cells as compared to control, unstimulated HT-29. As we previously described TUBB3 is upregulated during EMT in Snail-expressing CRC lines [22]. Thus, in the next set of studies, we silenced TUBB3 expression in TGF-β1-stimulated HT-29 cells both in the absence or presence of pcDNA-TUBB4B. According to previous analyses, we used 100 nM TUBB3 siRNA [22], and in each case, TUBB3 was silenced by approximately 0.75 (Figure S2A,B). Additionally, the Western blot analysis with antibodies recognizing other tubulin subunits (TUBB1, TUBB3, TUBB4B, TUBB6) confirmed the specificity of the siRNA sequences (Figure S3). Lowering TUBB3 expression resulted in a reduction of elongation ratio (1.5 times as compared to control) shifting the cell shape toward more rounded (Figure 3B). Simultaneous decrease of TUBB3 and increase of TUBB4B in the TGF-β1-stimulated HT-29 cells resulted in the decrease of elongation ratio that reached the same levels as control, unstimulated/pre-invasive HT-29 cells. Further confocal microscopy studies showed that TUBB4B presence affected the accumulation of microtubules (calculated as a green intensity level) in the basal parts of TGF-β1-stimulated HT-29 cells (Figure 3C). Silencing of TUBB3 (by siRNA) in TGF-β1-stimulated cells caused a similar accumulation and the cells were still loose, suggesting that the disruption of cell-cell interactions was a result of decreased TUBB4B level (Figure 3C). In agreement with this hypothesis, the analysis of green labeling intensity of upper scans of the cell showed about 0.5 lower green intensity in cells overexpressing TUBB4B. That effect was even stronger (0.75 decrease) in the cells with silenced TUBB3. The modulation of both tubulin subunit levels (TUBB3 silencing and TUBB4B overexpression) resulted in almost complete inhibition of the TGF-β1 effects (Figure 3B,C).

### 3.4. Changes in TUBB4B and TUBB3 Levels Modulate Microtubule Polymerization in TGF-β1 Stimulated HT-29 Cells

**Figure 4:**
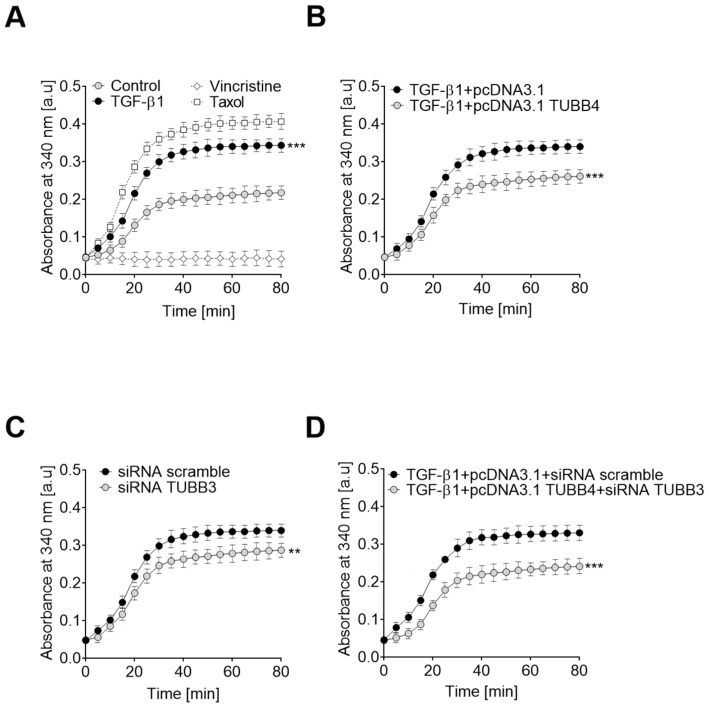
Modulations of TUBB4B and TUBB3 levels are critical for the rate of microtubule polymerization during EMT. The changes in microtubule polymerization were analyzed (**A**) in control and TGF-β1-stimulated HT-29 cells. Control cells were also treated with Taxol and vincristine-10 µM in both cases, (**B**) TGF-β1-stimulated HT-29 cells transfected with pcDNA and pcDNA/TUBB4B, (**C**), scramble and TUBB3 siRNA treated control HT-29 cells, and (**D**) both pcDNA/TUBB4B and siRNA TUBB3 treated TGF-β1-stimulated HT-29 cells. Results are plotted as the extent of tubulin polymerization (A340 nm) and a function of time and represent means of triplicate determinations at each time point. For graphic clarity, only data points from odd-numbered minutes are presented. ** *p* < 0.01, *** *p* < 0.005, relative to following controls: (A) non-stimulated cells TGF-β1-stimulated HT-29 cells transfected with empty pcDNA3.1 plasmid, (B) scramble siRNA treated HT-29 cells (C) or TGF-stimulated HT-29 treated with empty pcDNA3.1 plasmid and scramble siRNA (D); *n* = 5 * *p* < 0.05, *** *p* < 0.005.

Analysis of the polymerization/depolymerization ratio in control and TGF-β1 stimulated cells showed that induction of the EMT process correlates with faster polymerization of microtubules (Figure 4A). As controls, we used taxol and vincristine, two known agents that have the ability to block depolymerization or inhibit polymerization, respectively [32]. Increasing the level of TUBB4B in TGF-β1 stimulated cells by transfection with the plasmid carrying the TUBB4B gene resulted in a decrease of the polymerization ability of isolated microtubules (Figure 4B). Our previous experiments showing an increase of TUBB3 levels in EMT-induced cells prompted us to examine if alteration of the tubulin isoform levels also modulates polymerization of microtubules (Figure 4C). We found that prevention of TUBB3 expression by siRNA (Figure S2C) resulted in a decrease of microtubule polymerization. In spite of this, as in the presence of higher TUBB4B levels (Figure S4), the ability of microtubule polymerization was markedly weaker than observed in non-stimulated cells. Modulation of the levels of both tubulins (i.e., an increase of TUBB4B and decrease of TUBB3 levels) caused the substantial reduction of the microtubule polymerization capacity (Figure 4D). Therefore, we conclude that the change in polymerization capacity observed during EMT depends on the level of both tubulin isotypes.

### 3.5. Level of TUBB4B Determines the Microtubules-Vimentin Interaction in TGF-β1 Stimulated HT-29 Cells

**Figure 5:**
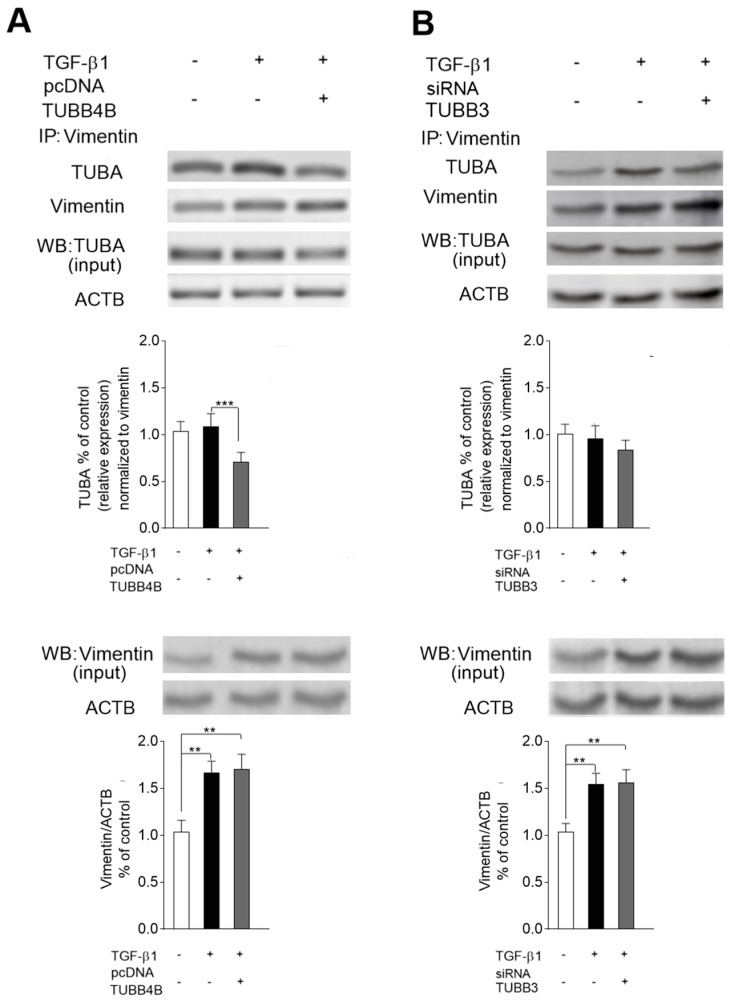
The presence of TUBB4B regulates the interaction of vimentin with tubulins in colon cancer cell lines undergoing EMT. The range of interaction between microtubules and vimentin in the cytoskeleton lysates of control and TGF-β1-treated HT-29 cells was evaluated by immunoprecipitation assay with rabbit rabbit-anti-human vimentin. The level of microtubules in precipitates was detected by Western blot with anti-human antibodies that recognize TUBA. Reversing of TUBB4B downregulation (**A**) and TUBB3 upregulation (**B**) was achieved by transfection with pcDNA/TUBB4B and treatment with siRNA/TUBB3, respectively. Additionally, levels of TUBA and vimentin were checked in the input samples. Protein level quantification was done as described in Figure 1. In the presented experiments vimentin (input) was used as a control. Error bars represent the SD from three independent experiments; *n* = 3; ** *p* < 0.01, *** *p* < 0.005 (relative to control samples). + with and – without as indicated in the Figure.

It has been established that the location of microtubules in mesenchymal cells is regulated by vimentin [21]. Thus, we analyzed the role of TUBB4B downregulation and TUBB3 upregulation during EMT, in the interaction between vimentin and microtubules in TGF-β1-stimulated HT-29 cells (Figure 5). To study the interaction of tubulins with vimentin, we performed the co-immunoprecipitation of the protein lysates obtained from control and TGF-β1-stimulated cells with an antibody against vimentin. It was followed by Western blot analysis with an antibody that binds TUBA. To check whether downregulation of TUBB4B in EMT is responsible for interaction between vimentin and microtubules, we restored the TUBB4B level by transfecting the TGF-β1-stimulated cells with pcDNA-TUBB4B (Figure S4). In such cells, we observed about 0.28 lower level of immunoprecipitated complex of TUBBA-vimentin (normalized to immunoprecipitated vimentin) in comparison to only TGF-β1 stimulated cells, suggesting that TUBB4B regulates the interaction of vimentin with tubulins. (Figure 5A). In contrast, reversing of TUBB3 upregulation by silencing with siRNA/TUBB3 (Figure S2D) did not affect the changes in the level of microtubule-vimentin complex (Figure 5B).

### 3.6. Decreased Level of TUBB4B in Microtubules Correlates with Increased Migration Towards Collagen I of TGF-β 1 Stimulated Colon Cancer Cell Lines

**Figure 6:**
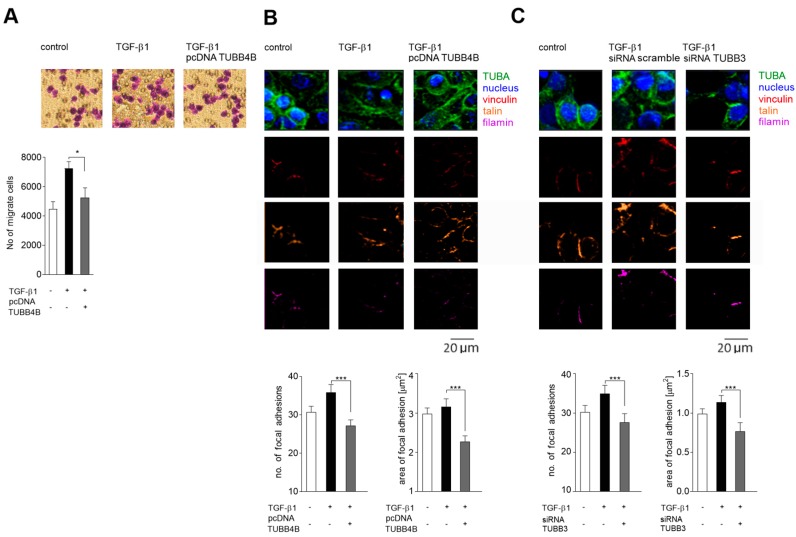
Modulation of TUBB3 and TUBB4B is critical for the regulation of cell migration through induction of focal adhesion maturation during EMT in colon cancer cells. (**A**) Transmigration of control (white bar), TGF-β1-stimulated (black bar), and TGF-β1-stimulated/pcDNA TUBB4B transfected (gray bar) - HT-29 cells towards collagen was determined. Error bars represent the SD from three independent experiments; *n* = 3; * *p* < 0.05. (**B**,**C**) show the number and size of focal adhesions that were calculated in control and in the TGF-β1-stimulated mock or pcDNA TUBB4B transfected cells and in the presence of scrambled and TUBB3 specific siRNA, respectively. Cells were stained with antibodies against vinculin connected with Alexa 633, talin connected with Alexa 543; filamin connected with cy5. The analysis of size and number of focal adhesions labeled with vinculin was made in ImageJ software on inverted colored pictures. Error bars represent the SD from three independent experiments; *n* = 3; *** *p* < 0.005. Statistical variability was measured relative to TGF-β1 stimulated cells. + with and – without as indicated in the Figure.

Restoration of TUBB4B levels in HT-29 cells undergoing EMT also reduced the ability of cell migration towards collagen (Figure 6A). The confocal microscopy of the TGF-β1 stimulated cells transfected with pcDNA TUBB4B (Figure S4) showed a decrease in the number and size of focal adhesion contacts (cells was labeled with rabbit antibodies recognized vinculin, filamin, and talin, specific focal adhesion markers). These contacts were usually smaller in TGF-β1 treated and cDNA TUBB4B transfected cells than only TGF-β1 stimulated cells (Figure 6B). We conclude that a decrease of TUBB4B level during EMT affects the disruption of vimentin-microtubules connection and promotes cell migration. This disruption was enhanced after TUBB3B silencing (Figure 6C) further suggesting that microtubule polymerization is regulated by TUBB3 and TUBB4B levels and influences cell migration by ensuring formation of focal adhesions.

## 4. Discussion

Microtubules (MT), together with microfilaments and intermediate filaments, are known to be engaged in cell cytoskeleton formation [33]. The modulation of the expression and post-translational modifications of particular tubulins that form polymers of MT were studied and described in the development of various cancers (for a review see: [7,34]). Nevertheless, the roles of individual tubulins and their subunits in cancer progression and in modulation of cell-cell contact are still poorly understood.

The data presented in these studies demonstrate for the first time that downregulation of TUBB4B and upregulation of TUBB3 observed during EMT is crucial for establishing the mesenchymal character of colon cancer cells. This involvement manifests itself in the regulation of the cell elongation and vimentin-microtubules interaction that is responsible for the cell polarity and increased cell migration. We show that all those processes are regulated by higher microtubule polymerization and are dependent on EMT-initiated modulations of both tubulins. 

Our study indicates that TUBB4B is highly expressed in unstimulated HT-29 cells and its level significantly decreases in EMT-induced cells. Accordingly, we observed a significantly lower level of TUBB4B protein in the LoVo cell line isolated from a patient diagnosed with an invasive stage of colon cancer (grade 3). Our observation suggests for the first time that the protein level of TUBB4B could be used as a marker for detection of the colon cancer cells that are in the early stage of invasiveness. We also found that while TUBB4B protein levels in EMT-induced HT-29 CRC cells dropped, gene expression remained only slightly decreased. 

It is important to note that modulations of the expression of tubulins, including TUBB4B, occur during tumor progression and contribute to cell survival and induction of chemoresistance [35]. In some solid and hematological tumors, the altered expression of TUBB3 and TUBB4B was observed both at the gene and the protein level [36]. The mechanism regulating expression consists of processes that affect gene and protein levels [37] and should be considered separately and related to the origin, primary location, and stage of the tumor. Perhaps the slight decrease of TUBB4 mRNA in EMT-induced cells (Figure 1) is the effect of a protective mechanism that allows the cell to more quickly return to the epithelial state during the reverse, mesenchymal-epithelial transition. The post-translational mechanisms that control tubulin expression in cancer cells on advanced stages are highly complex as well [33]. TUBB3, the best characterized subunit of the beta-tubulin, is generally upregulated during cancer progression. However, the TUBB2 and TUBB4B subunits can be up- or down-regulated, depending on the localization of the primary tumor [33]. Additionally, accumulation of particular beta-tubulins correlates with alterations in MT stability during cancer development [38]. The composition of tubulin subunits in MT modulate their polymerization ability [17,39]. It has been shown that TUBB4BTUBA1 or TUBB2TUBA1 dimers decrease the speed of microtubule polymerization, whereas microtubules composed on TUBB3TUBA1 are characterized by a higher dynamic of polymerization [40]. Previously we detected the influence of EMT on TUBB3 phosphorylation [22] but here we did not observe any modulations in the selected post-translational modifications of TUBB4B subunit. Both phosphorylation and glycosylation of TUBB4B remained unchanged after TGF-β1 stimulation. Therefore, we assume that they are not involved in the regulation of the EMT process. Additionally, in most of the tumors, the alterations in the level of some tubulin-subunits regulate the interaction between MT and microtubule-associated proteins [35]. This particular tubulin-subunit level change might further influence microtubule dynamics in a tubulin isotype-specific manner. Multiple studies have shown that overexpression of TUBB3 increase polymerization of microtubules while TUBB2 or TUBB4B has an opposite effect [38,41,42]. The collective influence of tubulin isotype composition and the microtubule-associated proteins that spatiotemporally interact with the MT network may regulate basal MT dynamics. 

We also focused here on the mechanism of how TUBB4B downregulation affects colon cancer cells to become more invasive. Our more detailed studies showed that in the cells that are undergoing EMT but have overexpressed TUBB4B, microtubules are reoriented and dispersed over the cell. In consequence, these cells lose their polarity. The same was observed, even to higher extent, in the cells where TUBB3 was also silenced. As the consequence of the disruption of microtubules’ polarity, the EMT-induced cells with the silenced TUBB3 and overexpressed TUBB4B became more round and resembled control, non-stimulated HT-29 cells. Additionally, we found that TUBB4B regulates the formation of focal adhesions. We observed that smaller focal adhesion correlates with reduction of cell migratory ability in EMT-induced HT-29 cells overexpressing the TUBB4B protein. Microscopic analysis of EMT-induced cells showed that microtubules were located mainly below the nucleus.

Vimentin is known to directly enhance cell migration [43,44]. Its upregulation observed in invasive cancer stages ensures mechanical homeostasis in a cancer cell [45]. Critical for the mechanical cell integrity is the interaction between vimentin and microtubules. This interaction is required for vimentin function as a linker between microtubules and actin [46]. Our studies show that as a result of TUBB4B protein decrease, which causes microtubule decomposition, the interaction between vimentin and microtubules is augmented in the invasive colon cancer cells. Thus, we propose that modulation of the TUBB4B protein is critical for vimentin-microtubules interaction in invasive colon cancer cells. Vimentin is also regarded as a modulator of cell polarity and motility by maintaining the cytoskeleton structure and stability of mechanical force [21], and is involved in the proper cell tension and mechanotransduction and counterforce to deformation generated by the microenvironment [47,48]. Based on this reasoning, we posit that modulation of beta-subunits composition by increasing of TUBB4B in colon cancer cells undergoing EMT can affect microtubule colocalization with vimentin. As a result, the cells will stay elongated, as this process is dependent on vimentin-microtubules interaction, focal adhesion will be destabilized, and cell migration ability will be inhibited.

## 5. Conclusions

**Figure 7:**
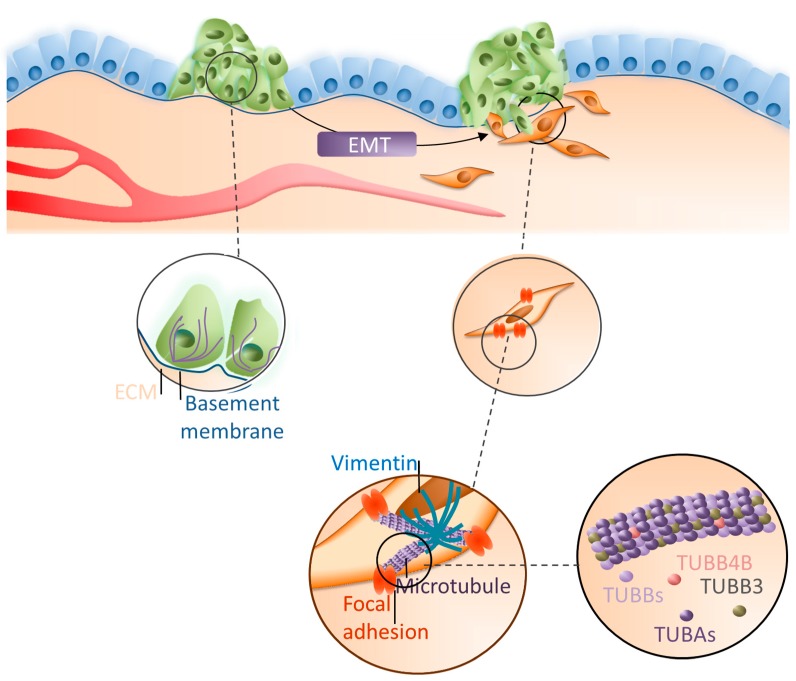
Modulation of TUBB3 and TUBB4B during EMT in colon cancer cells. During EMT epithelial cancer cells (green cells) become elongated (orange cells) and migrate faster as the result of microtubule reorganization. The process is dependent on the decrease of TUBB4B levels and the increase of TUBB3 levels. Changes in the microtubule subunit composition modulate the cell polarization and control the location and function of vimentin as well as the focal adhesion site to that observed in mesenchymal cells.

We uncovered that decomposition of microtubules associated with the decrease of TUBB4B protein observed during EMT is critical for the regulation of cell polarization and control of vimentin localization and function (Figure 7). 

Additionally, an increase of TUBB3 and decrease of TUBB4B by affecting microtubule polymerization regulates cell elongation and migration, two critical processes characteristic for mesenchymal cells. Hence, we suggest that regulation of the TUBB4B subunit may become a new target to inhibit the invasion of colon cancer, and perhaps other cancers where TUBB4B is downregulated during tumor progression to the metastatic stages.

## Supplementary Materials

The following are available online at https://www.mdpi.com/2073-4409/8/8/810/s1, Figure S1: The level of TUBB4B in colon cancer cells correlated with cancer progression, Figure S2: The effectivity of TUBB3 downregulation in individual experiments., Figure S3: Confirmation the TUBB3 and TUBB4B antibody specificity, Figure S4: The effectivity of TUBB4B upregulation in individual experiment.
