# Supplementary material for: TUBB4B Downregulation Is Critical for Increasing Migration of Metastatic Colon Cancer Cells
Source: Cells. 2019 Aug 1;8(8):810. doi: 10.3390/cells8080810 (PMC6721557; doi:10.3390/cells8080810)
Supplement: Supplementary file 1 [file cells-08-00810-s001.pdf]

Supplementary data

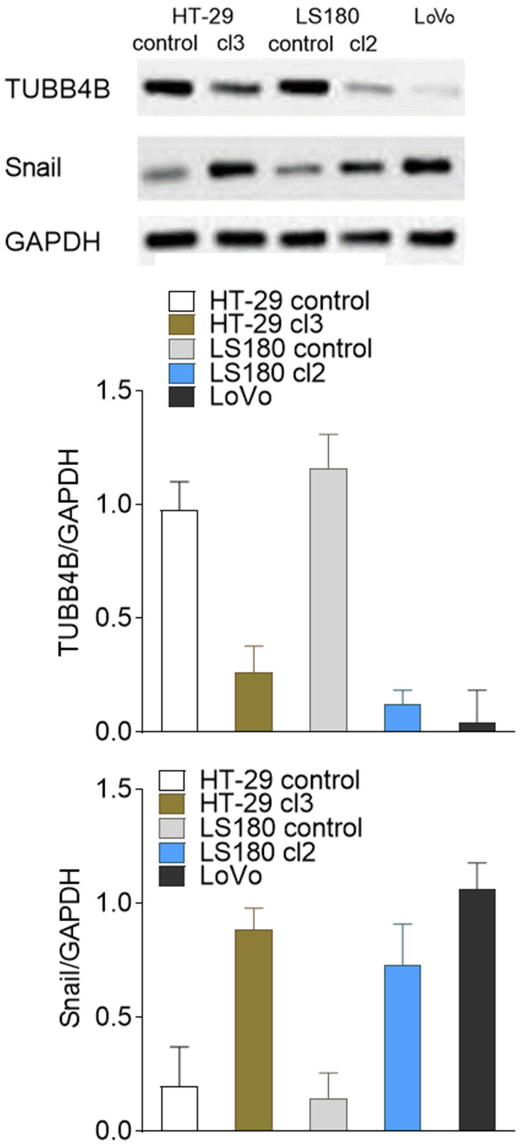

Figure S1. The level of TUBB4B in colon cancer cells correlated with cancer progression. The expression of TUBB4B and Snail were analyzed in empty vector- and Snail-transfected HT-29 (cl3) and LS180 (cl2) clones and LoVo cells. The protein level in the whole cell lysates were evalulated by Western blot assay using mouse monoclonal anti-TUBB4B and anti-snail antibodies. The quantity of the protein level was normalized to GAPDH. N = 3.

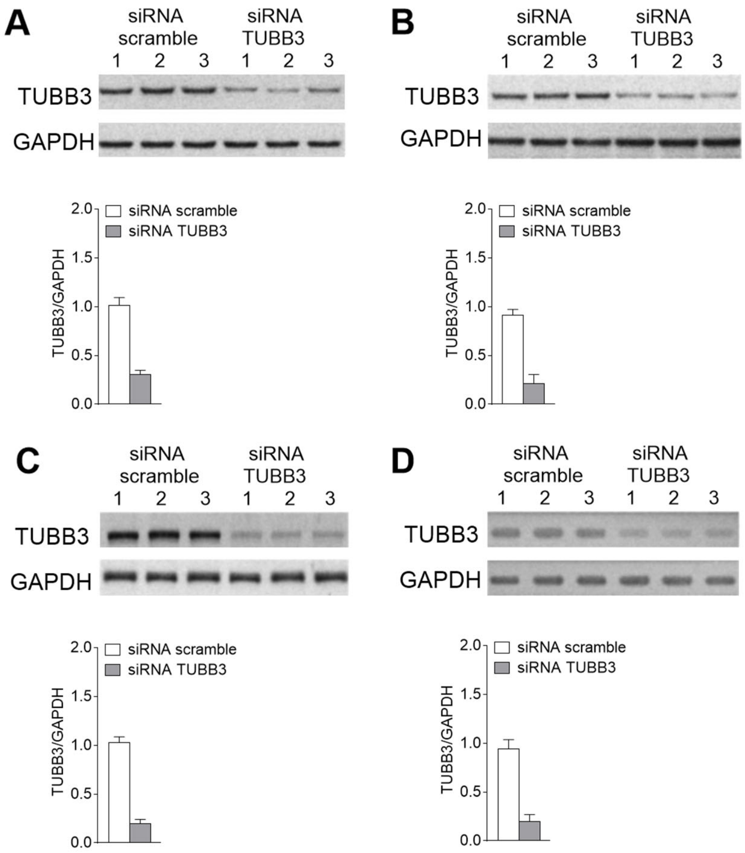

Figure S2. The efectivity of TUBB3 downregulation in invidual experiments. Based on our previous studies [Sobierajka K. et al. BBA 2016, 1863, 2221–2233] 100nM of specific siRNA was chosen to downregulate TUBB3 protein level. In each experiment the efectivity TUBB3-silencing was analyzed by Western blot assay with mouse monoclonal antibodies specifically recognized TUBB3. The quantity of TUBB3 protein level was normalized to GAPDH. N = 3**.**

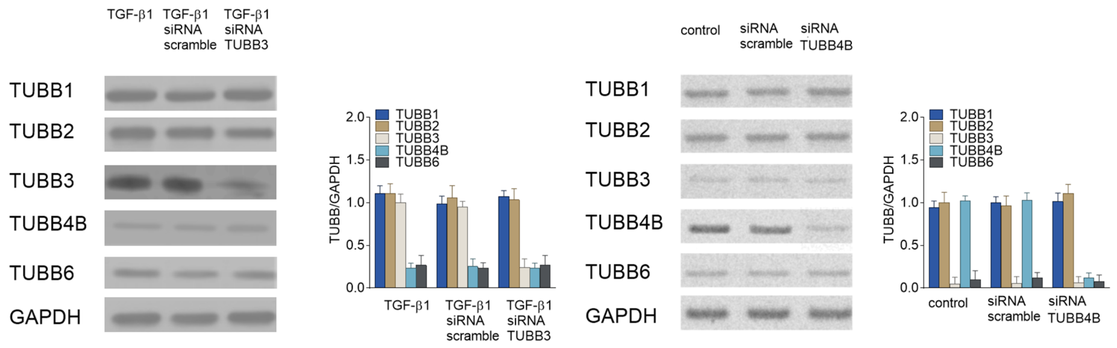

Figure S3. Confirmation the TUBB3 and TUBB4B antibody specifity. After (A) TUBB3 and (B) TUBB4B downregulation with 100nM of specific siRNA the level of TUBB1, TUBB2, TUBB3, TUBB4B and TUBB6 was analyzed by Western blot assay with appropriate mouse monoclonal antibodies specifically recognized each tubulin subunits. The quantity of the protein level was normalized to GAPDH. N = 3.

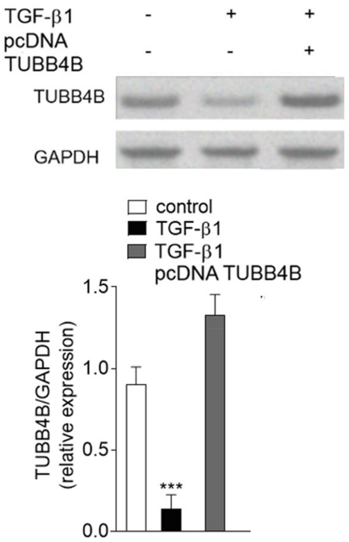

Figure S4. **The efectivity of TUBB4B upregulation in invidual experiment.** The TUBB4B upregulation in TGF-β1 stimulated cell was induce by transfection with pcDNA3.1 plasmid with TUBB4B. The level of TUBB4B upregulation was analyzed by Western blot assay with antibodies recognized TUBB4B. The quantity of the TUBB4B was normalized to GAPDH. N = 3.
